# Declining yet persistent asthma mortality among U.S. adults aged 65 years and older (1999–2020)

**DOI:** 10.3389/fmed.2026.1832774

**Published:** 2026-08-21

**Authors:** Tong Feng, Pengcheng Zheng, Yue Cao, Ding Han, Ran Duan

**Affiliations:** 1 Department of Respiratory and Critical Care Medicine, Deyang People's Hospital, Affiliated Hospital of Chengdu University of Traditional Chinese Medicine (TCM), Deyang, China; 2 School of Clinical Medicine, Chengdu Medical College, Chengdu, China; 3 Department of Respiratory and Critical Care Medicine, The First Affiliated Hospital of Chengdu Medical College, Chengdu, China; 4 Laboratory of Geriatric Respiratory Diseases of Sichuan Higher Education Institute, Chengdu, China; 5 Department of Cardiology, Affiliated Hospital of Southwest Medical University, Luzhou, China; 6 Department of Oncology, The First Affiliated Hospital of Chengdu Medical College, Chengdu, China

**Keywords:** asthma, older adults, health disparities, mortality trends, United States

## Abstract

**Background:**

Asthma in older adults (≥65 years) represents a growing yet understudied public health burden. Comprehensive spatiotemporal trends and future projections of asthma mortality in this population remain incompletely characterized. This study aimed to characterize the temporal, demographic, and geographic patterns of asthma mortality among U.S. adults aged ≥65 years, project future mortality trends through 2030, and evaluate state-level disparities relative to an empirical mortality frontier.

**Methods:**

Using the Centers for Disease Control and Prevention Wide-ranging Online Data for Epidemiologic Research (CDC WONDER) Underlying Cause of Death data from 1999 to 2020, we examined asthma mortality (ICD-10 J45–J46) among U.S. adults aged ≥65 years. Age-adjusted mortality rates (AAMRs) per 100,000 population were calculated. Joinpoint regression was used to characterize temporal changes and estimate annual percent changes (APCs). A non-seasonal ARIMA model was used to project mortality through 2030. State-level ecological associations with the Socio-demographic Index (SDI) and variation relative to an SDI-conditioned LOESS mortality frontier were evaluated.

**Results:**

Between 1999 and 2020, 38,101 asthma-related deaths occurred among adults aged ≥65 years, yielding an overall AAMR of 4.14 (95% CI, 4.09–4.18) per 100,000 population. Mortality declined by 54.5%, from 7.01 per 100,000 in 1999 to 3.19 per 100,000 in 2020. In the overall population, the decline slowed after 2010 (APC, −5.751% during 1999–2010 and −2.194% during 2010–2020). Sex-specific patterns differed: the female decline slowed after 2010, whereas the male decline slowed after 2008 and ceased after 2016. Women had a higher pooled AAMR than men (4.94 vs. 2.91 per 100,000), although the absolute difference between annual sex-specific rates decreased over time. Substantial geographic and racial/ethnic variation persisted. Overall AAMR point estimates were projected to range from 3.02 to 3.46 per 100,000 during 2021–2030. The LOESS-based frontier analysis showed considerable state-level variation in the distance between observed mortality and the empirical mortality benchmark.

**Conclusion:**

Although asthma mortality among U.S. adults aged ≥65 years declined substantially between 1999 and 2020, the decline slowed after 2010, and marked demographic and geographic disparities persisted. These population-level patterns identify groups and regions that may benefit from enhanced surveillance and further investigation. Because the analyses were based on aggregated observational data, the reported associations should not be interpreted as evidence of individual-level or state-level causal relationships.

## Graphical Abstract

**Figure:**
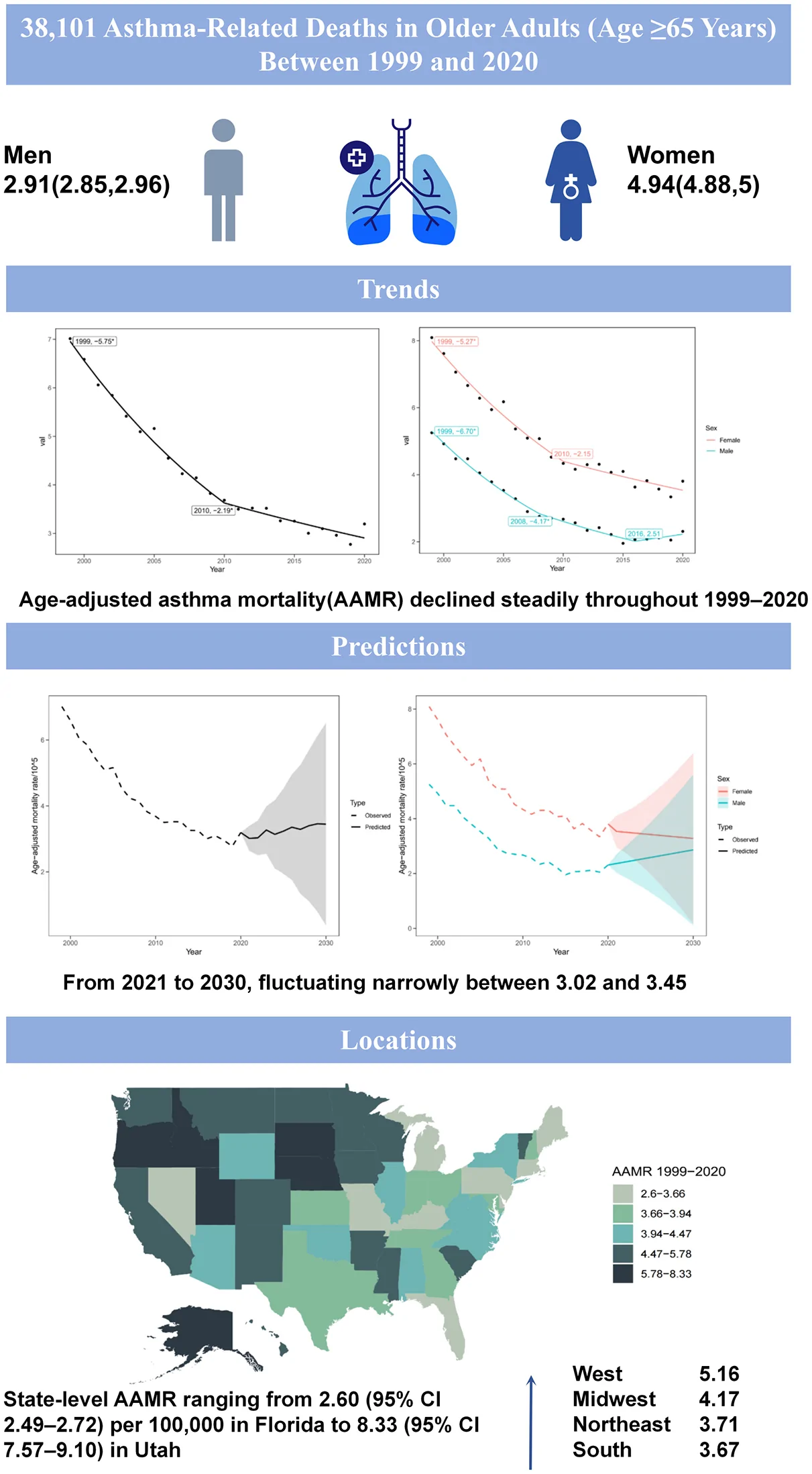

## Introduction

Asthma persists as a major global cause of preventable morbidity and mortality. According to the 2026 Global Initiative for Asthma (GINA) report, asthma is a common chronic respiratory disease, with prevalence varying substantially across countries and affecting approximately 1%−29% of the population (1). Among older adults, asthma represents a particularly important clinical and public health challenge because of age-related physiological changes, accumulated environmental exposures, and the high prevalence of comorbidities. The American Thoracic Society defines asthma in older adults as asthma in individuals aged ≥65 years, including both early-onset disease persisting into advanced age and late-onset disease diagnosed at or after age 65 (2). Epidemiologically, asthma incidence across the lifespan follows a U-shaped pattern, with older adults representing a second peak after childhood (3). Clinical presentation and management are complicated by declining lung function, cumulative environmental exposures, and a high prevalence of comorbidities (4, 5), which may be associated with more refractory symptoms and greater difficulty achieving asthma control (6). U.S. surveillance data showed that asthma prevalence among adults aged ≥65 years increased from 6.0% in 2001 to 8.1% in 2010, and this group had the second-highest hospitalization rate after children aged 0–4 years (7). The U.S. population aged ≥65 years is projected to increase from approximately 56 million in 2020 to 95 million by 2060, while the population aged ≥85 years is projected to increase from 6 million to 19 million (8). These demographic trends reinforce the growing public health relevance of asthma in older populations (9).

Similar declining trends in asthma-related healthcare burden have also been observed in other countries. In Brazil, a nationwide epidemiological analysis of the Unified Health Care System from 2008 to 2021 demonstrated substantial reductions in asthma-related hospitalizations and deaths over time, although a considerable residual burden persisted, with more than 55,000 hospitalizations annually and approximately one asthma-related death per day (10). These findings underscore that, despite improvements in asthma prevention and management, asthma continues to impose a substantial and potentially reducible healthcare burden.

Previous investigations using CDC WONDER mortality data have predominantly relied on conventional epidemiological methods, particularly Joinpoint regression, to characterize temporal trends in asthma mortality (11–13). These studies identified periods of accelerated decline that coincided with the broader adoption of controller therapies, reductions in smoking prevalence, and changes in public health practices. However, their ecological designs do not establish whether these population-level changes caused the observed mortality trends. Furthermore, previous studies have generally focused on historical patterns and have rarely incorporated time-series forecasting or state-level benchmarking methods.

Clarifying the demographic, socioeconomic, and geographic distribution of asthma-related deaths is important for identifying high-risk populations and supporting timely, targeted investigation. Population-level characterization may help public health authorities prioritize surveillance and resource planning for vulnerable groups, including older women, racial/ethnic minority populations, residents of rural or socioeconomically disadvantaged areas, and states with persistently elevated mortality. It may also help inform evaluation of strategies such as enhanced case detection, optimization of inhaled corticosteroid/long-acting β2-agonist use, smoking-cessation programs, and air-quality improvement measures (14).

The present study addresses these gaps by characterizing asthma mortality among U.S. adults aged ≥65 years from 1999 through 2020 using CDC WONDER data. We applied Joinpoint regression to identify changes in temporal trends, ARIMA models to project mortality through 2030, and SDI correlation and LOESS-based frontier analyses to describe state-level variation relative to an empirical mortality benchmark. The analyses were intended to identify temporal, demographic, socioeconomic, and geographic patterns rather than to estimate causal effects. By describing these population-level patterns, this study may help inform future surveillance, hypothesis generation, and the design of individual-level or policy-evaluation studies.

## Materials and methods

### Data sources

Mortality data were obtained from the Centers for Disease Control and Prevention Wide-ranging Online Data for Epidemiologic Research (CDC WONDER) Underlying Cause of Death, 1999–2020, by Bridged-Race Categories database. This database uses bridged-race population estimates and assigns decedents reporting multiple races to one of four mutually exclusive categories: White, Black or African American, American Indian or Alaska Native, and Asian or Pacific Islander. Asthma deaths among U.S. adults aged ≥65 years were identified using ICD-10 codes J45–J46, and AAMRs were calculated per 100,000 population using the 2000 U.S. standard population.

The primary analytic period was retained as 1999–2020 to maintain a consistent race-classification system and population-denominator framework across national, demographic, geographic, and race-stratified analyses. Beginning with the newer CDC WONDER Underlying Cause of Death, 2018–2024, by Single-Race Categories release, Asian and Native Hawaiian or Other Pacific Islander populations are reported separately, and persons reporting multiple races are retained as a distinct category rather than reassigned to one bridged-race category. Consequently, post-2020 observations were not directly appended to the primary race-stratified time series. Because this study used publicly available, de-identified, and aggregated data and involved no identifiable individual-level information, approval from a research ethics committee and informed consent were not required.

### Study design and population

This ecological, repeated cross-sectional study assessed spatiotemporal patterns in asthma-related mortality among U.S. adults aged ≥65 years from 1999 to 2020. The analyses were based on aggregated mortality records and focused on variation by demographic characteristics, geographic location, and calendar year rather than on individual-level exposures or outcomes. A schematic overview of the study design and analytical framework is presented in Supplementary Figure 1.

The study population included adults aged ≥65 years in all U.S. states and the District of Columbia. Mortality data were stratified by sex, age group (65–74, 75–84, and ≥85 years), calendar year, race/ethnicity, urbanization level, census region, and state, where available. This stratified design allowed us to characterize heterogeneity in mortality rates across population subgroups. Because the data were aggregated, observed differences between groups represent population-level associations and should not be interpreted as causal effects of sex, age, race/ethnicity, residence, socioeconomic conditions, or healthcare characteristics.

To reduce confounding from differences in age distributions across years and populations, AAMRs per 100,000 population were calculated using the 2000 U.S. standard population as the reference. This standardization supports comparisons across years and subgroups but does not eliminate confounding from unmeasured individual-level or contextual characteristics.

### Statistical analysis

#### Joinpoint regression analysis

Joinpoint regression was used to characterize statistically significant changes in AAMR trends from 1999 to 2020. The method identifies time points at which the slope of the temporal trend changes and estimates the APC for each segment (15, 16). The analysis was used to describe the timing and magnitude of changes in mortality trends. It was not designed to evaluate the effectiveness of specific public health interventions or to determine the causes of the observed changes.

#### ARIMA forecasting analysis

Annual overall AAMRs from 1999 to 2020 were modeled as a non-seasonal time series with one observation per year. Model identification and selection were performed using the auto.arima() function in the R forecast package. Candidate combinations of autoregressive order (*p*), differencing order (d), and moving-average order (q) were systematically evaluated. Because the observations were annual and no within-year seasonal pattern was present, seasonal ARIMA components were not included (17, 18).

Stationarity was formally assessed using the Kwiatkowski–Phillips–Schmidt–Shin test. The original series was non-stationary (KPSS statistic = 0.828; *P* < 0.01), and the first-differenced series remained non-stationary (KPSS statistic = 0.764; *P* < 0.01). After second-order differencing, the null hypothesis of stationarity was not rejected (KPSS statistic = 0.109; *P* > 0.10); therefore, *d* = 2 was used. The Bayesian Information Criterion was specified as the primary model-selection criterion, with the model having the lowest BIC selected as the final model. AIC and corrected AIC were also reported.

The optimal model was ARIMA (2, 2, 0), expressed as (1 – ϕ1*B* – ϕ2*B*^2^) (1 – *B*)^2^*Yt* = ε*t*, where B denotes the backshift operator and εt represents the residual. The selected model had AIC = −3.266, AICc = −1.766, and BIC = −0.279. Fitted and observed values were compared using Pearson's correlation coefficient. Model adequacy was evaluated using residual Q–Q, ACF, and PACF plots and the Ljung–Box test. Forecasts for 2021–2030 were generated with 95% prediction intervals.

#### SDI correlation analysis

The ecological association between state-level SDI and AAMR was evaluated using correlation analyses. These analyses quantified whether states with different levels of socioeconomic development also differed in asthma mortality at the population level. They did not estimate the causal effect of socioeconomic conditions on mortality and did not permit inference regarding associations at the individual level.

#### SDI-conditioned frontier analysis using LOESS

An SDI-conditioned frontier analysis was performed to evaluate potential room for improvement in state-level asthma mortality relative to the lowest empirically observed mortality rates at comparable levels of sociodemographic development. This approach was conceptually distinct from conventional stochastic frontier production analysis. It did not assume that SDI was a healthcare input and did not decompose residual variation into statistical noise and technical inefficiency.

The SDI was used as a contextual benchmarking variable because it summarizes broad differences in income, educational attainment, and fertility associated with population health. It was not considered a substitute for direct measures of healthcare resources or quality, such as specialist density, hospital or intensive-care capacity, insurance coverage, medication access, or adherence to asthma-management guidelines.

Let Yit denote the AAMR for state *i* in year *t*, and let Sit denote the corresponding SDI. Empirically best-performing observations along the lower boundary of the SDI–mortality distribution were used to construct the frontier. A locally estimated scatterplot smoothing model was fitted to these boundary observations. At each SDI value s, the local polynomial coefficients were estimated by minimizing minβ0, …, β*p*Σ*jwj*(*s*)[*Yj*−Σ*k* = 0*p*β*k*(*Sj*−*s*)*k*]^2^, where *wj*(*s*) was a distance-based LOESS weight, *p* was the local polynomial degree, and the fitted intercept β0(*s*) represented the estimated frontier $\hat{f}$(*s*).

The absolute frontier gap was calculated as Git = max[0, Yit – $\hat{f}$(Sit)]. A larger frontier gap indicated a greater difference between the observed mortality rate and the lowest empirically observed mortality benchmark among observations with comparable SDI values. The gap was interpreted as potential room for mortality reduction conditional on SDI and not as a direct measure of health-system inefficiency or a causal estimate of preventable mortality.

## Results

## Demographic and geographic disparities in asthma mortality

From 1999 to 2020, 38,101 asthma-related deaths occurred among U.S. adults aged ≥65 years, yielding an overall AAMR of 4.14 (95% CI, 4.09–4.18) per 100,000 population (Table 1). Mortality was highest in the West Census Region (AAMR, 5.16; 95% CI, 5.06–5.26) and lowest in the South and Northeast (3.67 and 3.71, respectively). Women had a higher pooled AAMR than men (4.94 vs. 2.91 per 100,000). No meaningful difference was observed by Hispanic ethnicity.

**Table 1:** Demographic characteristics of deaths due to asthma in the elderly population in the United States from 1999 to 2020.

Characteristic	Deaths/ population	AAMR (95 %CI)
Overall	38,101/928,476,665	4.14 (4.09, 4.18)
Census region		
Midwest	8,856/207,118,111	4.17 (4.08, 4.25)
Northeast	6,842/179,714,300	3.71 (3.62, 3.8)
South	12,349/343,442,253	3.67 (3.61, 3.74)
West	10,054/198,202,001	5.16 (5.06, 5.26)
Sex		
Female	27,384/527,632,126	4.94 (4.88, 5)
Male	10,717/400,844,539	2.91 (2.85, 2.96)
Hispanic origin		
Hispanic or Latino	2,484/65,124,722	4.12 (3.96, 4.29)
Not Hispanic or Latino	35,497/863,351,943	4.1 (4.05, 4.14)
Race		
American Indian or Alaska Native	208/6,051,868	3.84 (3.3, 4.38)
Asian or Pacific Islander	1,940/35,919,615	6.07 (5.8, 6.34)
Black or African American	5,093/83,274,492	6.29 (6.12, 6.47)
White people	30,860/803,230,690	3.78 (3.74, 3.83)
Urbanization		
Metro	30,666/760,496,648	4.05 (4, 4.1)
Non-metro	7,435/167,978,547	4.5 (4.4, 4.6)

AAMR, age-adjusted mortality rate; CI, confidence interval.

By race, Black (AAMR, 6.29) and Asian/Pacific Islander (6.07) older adults had the highest mortality, whereas White older adults had the lowest AAMR (3.78). Asthma mortality was higher in non-metropolitan than metropolitan areas (4.50 vs. 4.05 per 100,000).

**Figure 1:**
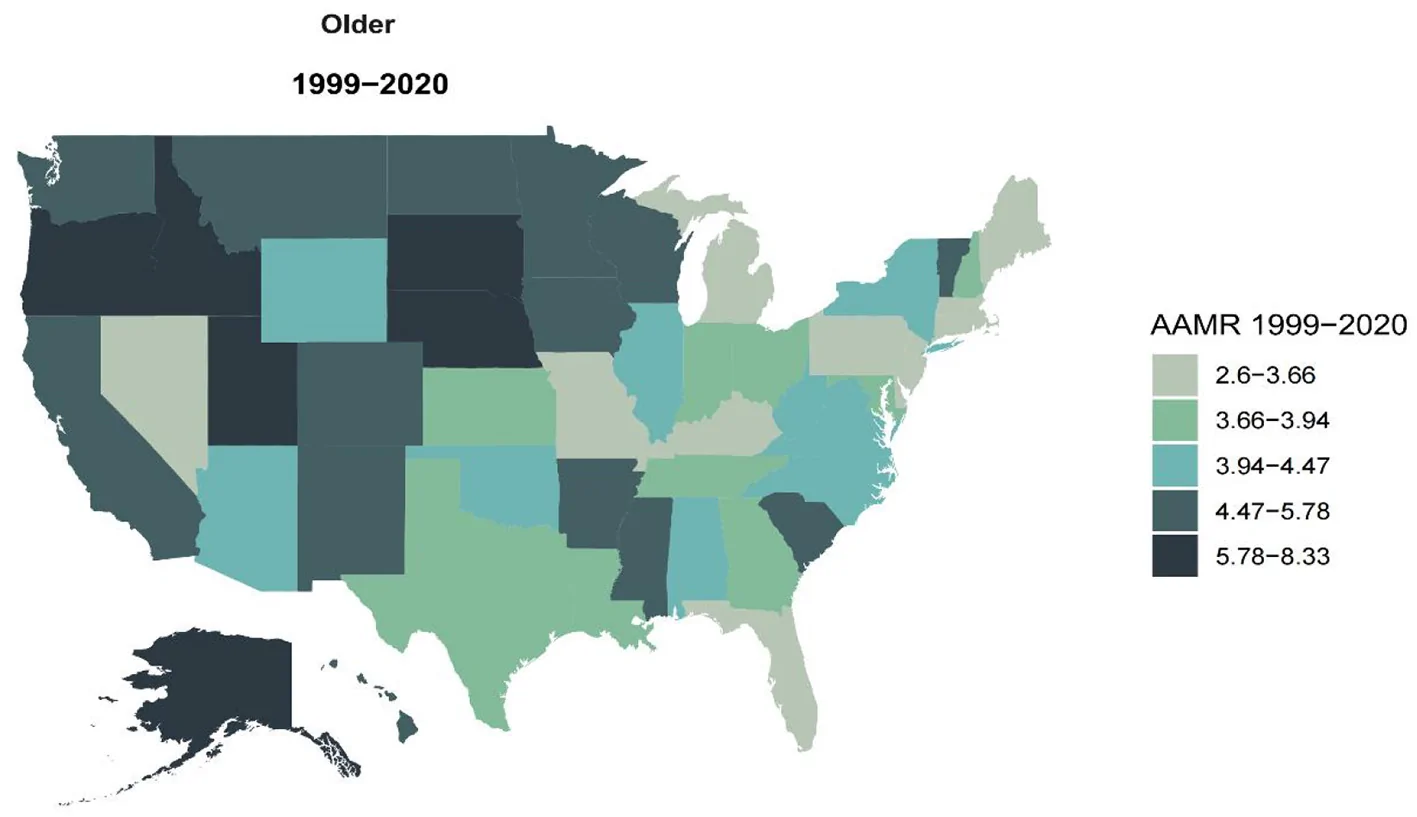
Age-adjusted asthma mortality rates (AAMR) per 100,000 older adults by U.S. state, 1999–2020.

State-level AAMRs varied widely, ranging from 2.60 per 100,000 in Florida (95% CI, 2.49–2.72) to 8.33 per 100,000 in Utah (95% CI, 7.57–9.10; Figure 1 and Supplementary Table 1). The highest rates were observed in Utah, Nebraska, South Dakota, Idaho, Oregon, Minnesota, Hawaii, Alaska, and Vermont (all >5.5 per 100,000), whereas lower rates occurred in Florida, Nevada, Rhode Island, Delaware, Pennsylvania, New Jersey, and Michigan. California and New York together accounted for nearly 20% of total U.S. asthma deaths in this age group.

## Sex- and age-specific trends in mortality rates

**Figure 2:**
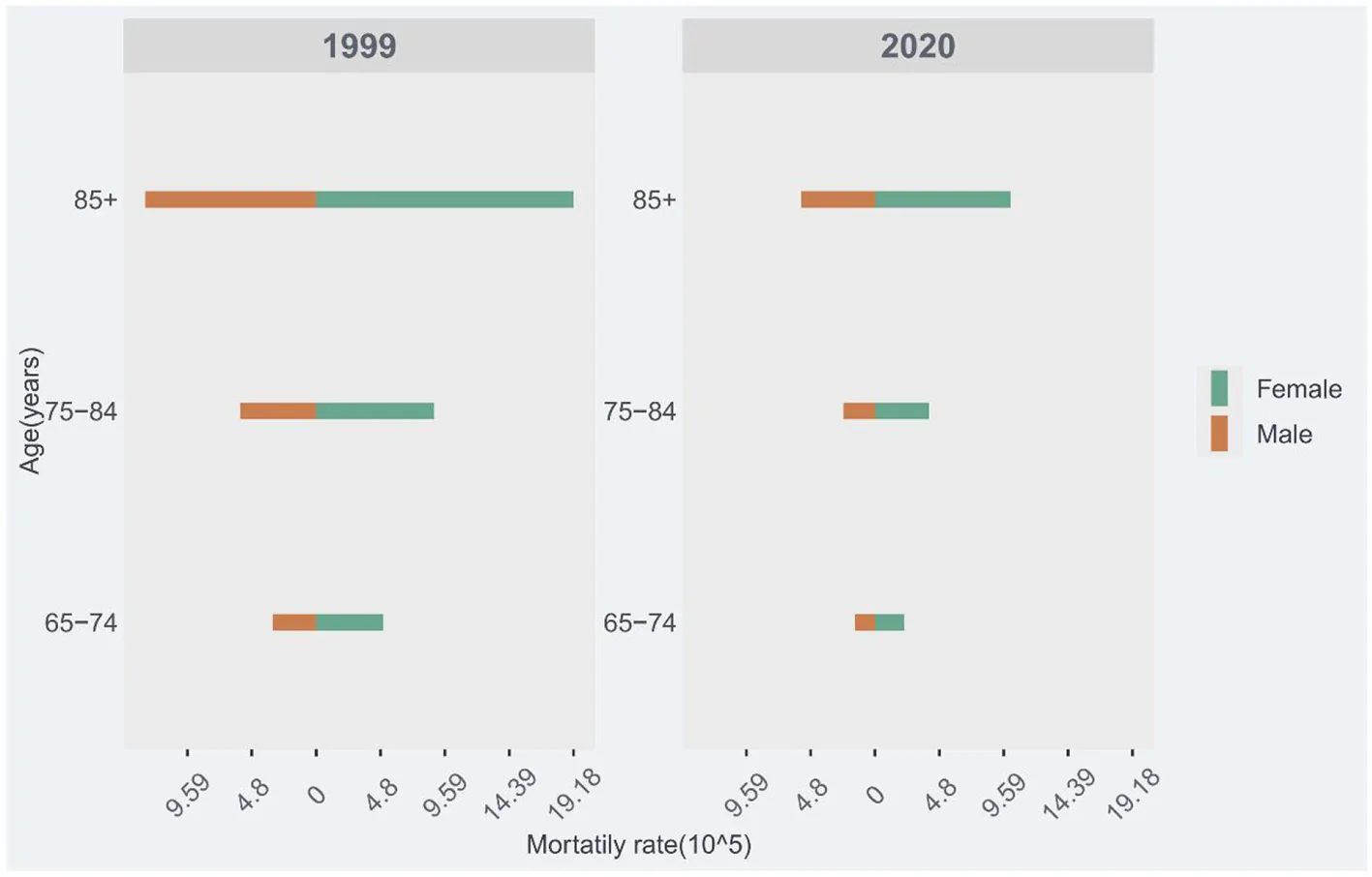
Sex-specific asthma mortality rates by age group in 1999 and 2020.

**Figure 3:**
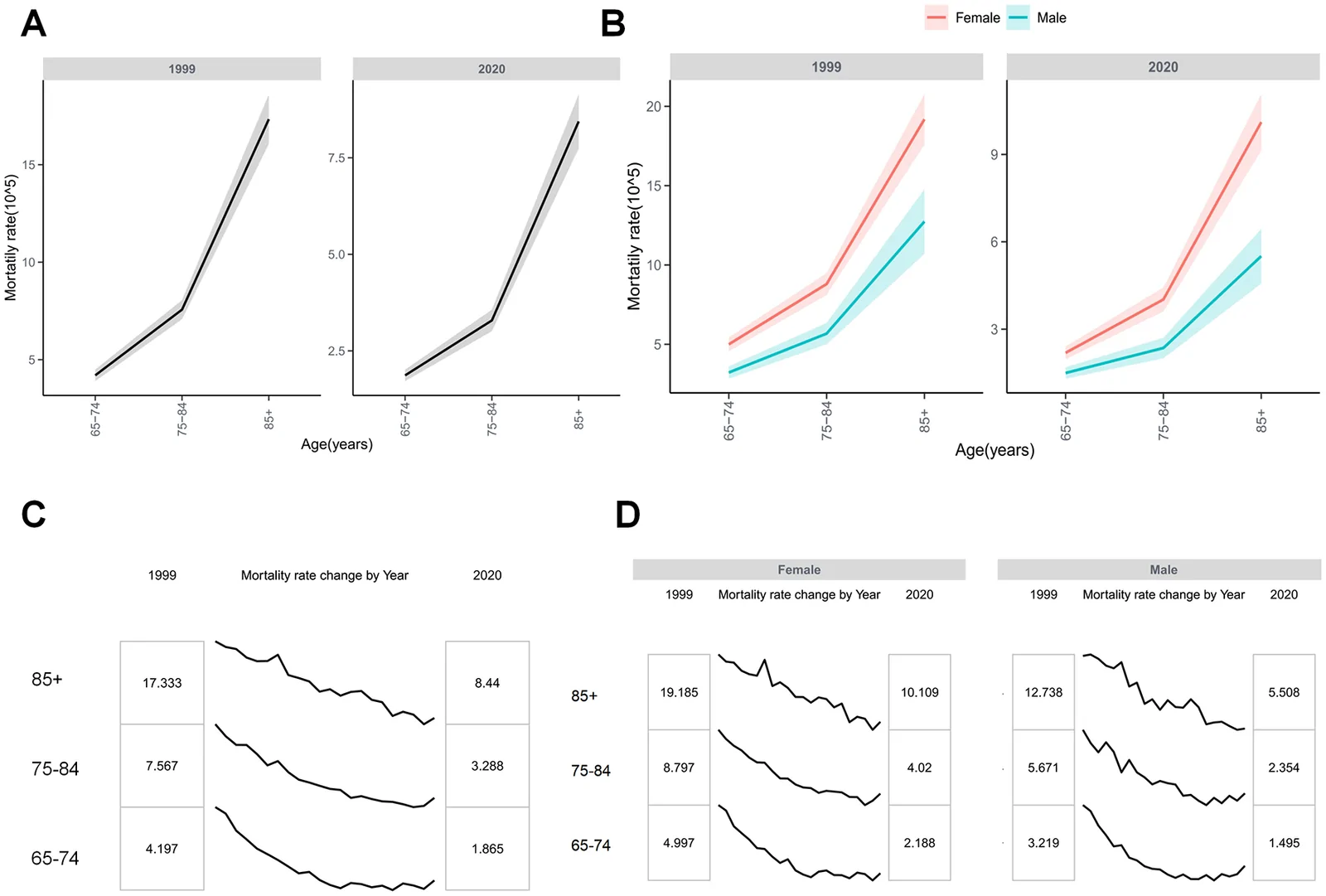
Age- and sex-specific asthma mortality rate trends and annual changes, 1999–2020. **(A)** Overall rates by age group in 1999 and 2020. **(B)** Sex-stratified rates by age group in 1999 and 2020. **(C)** Annual mortality rate change by age group. **(D)** Annual mortality rate change by sex and age group.

Age- and sex-specific asthma mortality rates changed markedly from 1999 to 2020 (Figures 2, 3, Supplementary Table 2, and Supplementary Figure 2). In the total population, rates decreased from 4.20 to 1.87 per 100,000 among adults aged 65–74 years, from 7.57 to 3.29 among those aged 75–84 years, and from 17.33 to 8.44 among those aged ≥85 years. The largest absolute reduction was observed in the ≥85-year age group.

Among women, the corresponding rates decreased from 5.00 to 2.19 per 100,000 in those aged 65–74 years, from 8.80 to 4.02 in those aged 75–84 years, and from 19.19 to 10.11 in those aged ≥85 years. Among men, rates decreased from 3.22 to 1.50, from 5.67 to 2.35, and from 12.74 to 5.51 per 100,000 in the three age groups, respectively. Female rates exceeded male rates in all age groups at both time points, although the absolute sex difference narrowed by 2020.

## Long-term trends and age distribution of asthma burden

**Figure 4:**
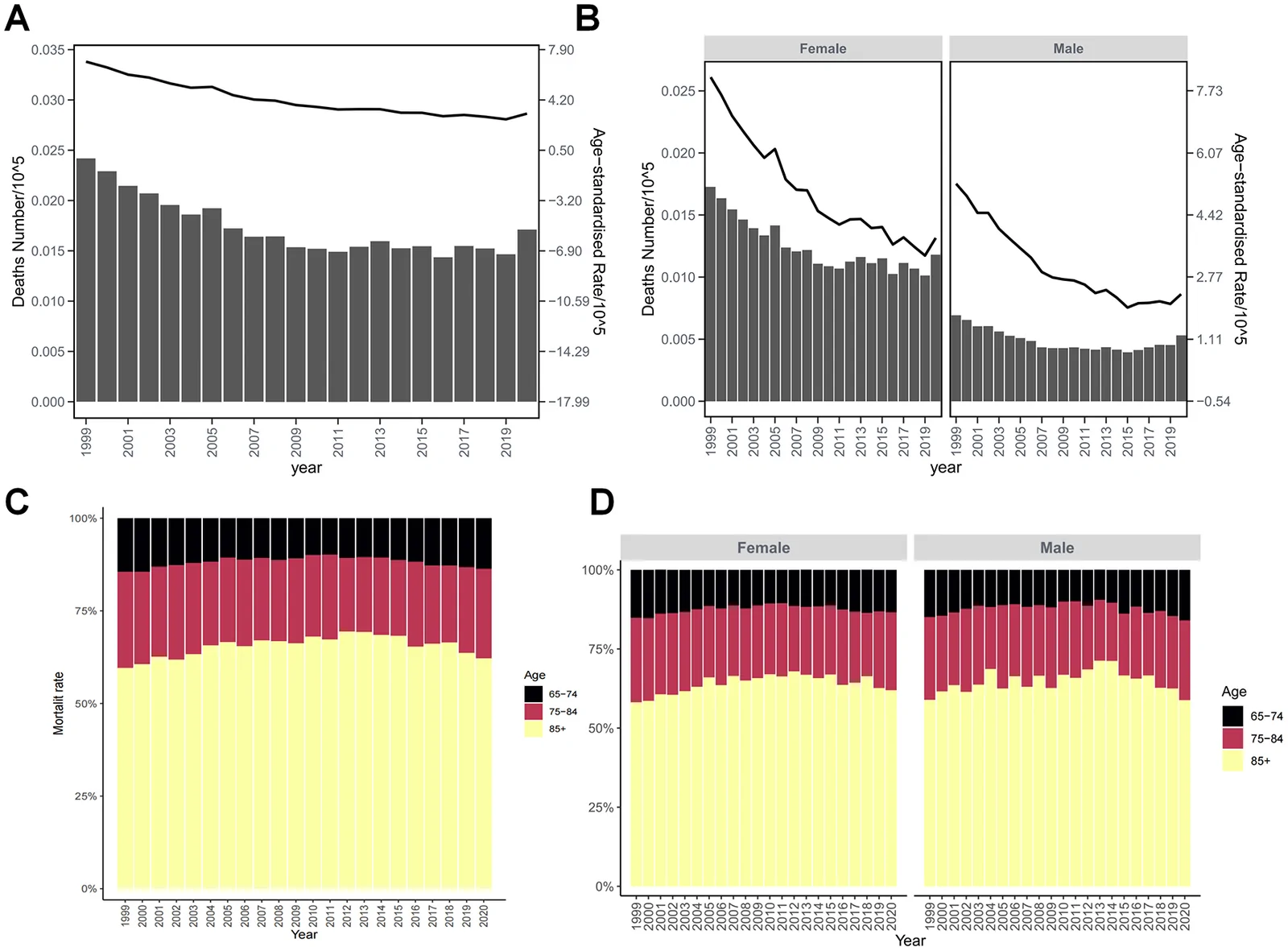
Long-term trends in asthma mortality burden and age distribution, 1999–2020. **(A)** Annual asthma deaths population and Age-adjusted asthma mortality. **(B)** Annual asthma deaths population and Age-adjusted asthma mortality by sex. **(C)** Proportion of asthma deaths by age group. **(D)** Age-specific mortality contribution by sex.

The observed AAMR decreased from 7.014 per 100,000 in 1999 to 3.194 per 100,000 in 2020, corresponding to a cumulative reduction of 54.5% (Figure 4A and Supplementary Table 3). Female AAMR decreased from 8.091 to 3.808 per 100,000, a 52.9% reduction, whereas male AAMR decreased from 5.253 to 2.310 per 100,000, a 56.0% reduction (Figure 4B). The absolute female–male difference narrowed from 2.838 to 1.498 per 100,000. Annual asthma-related deaths decreased from 2,418 to 1,710, representing a 29.3% reduction. This comparatively smaller decline in the absolute number of deaths occurred during substantial growth and aging of the U.S. older adult population; however, the analysis did not formally decompose changes in death counts into demographic and rate-related components. The age composition of asthma deaths also shifted over time (Figures 4C, D).

## Joinpoint analysis and sex-specific inflection points

**Figure 5:**
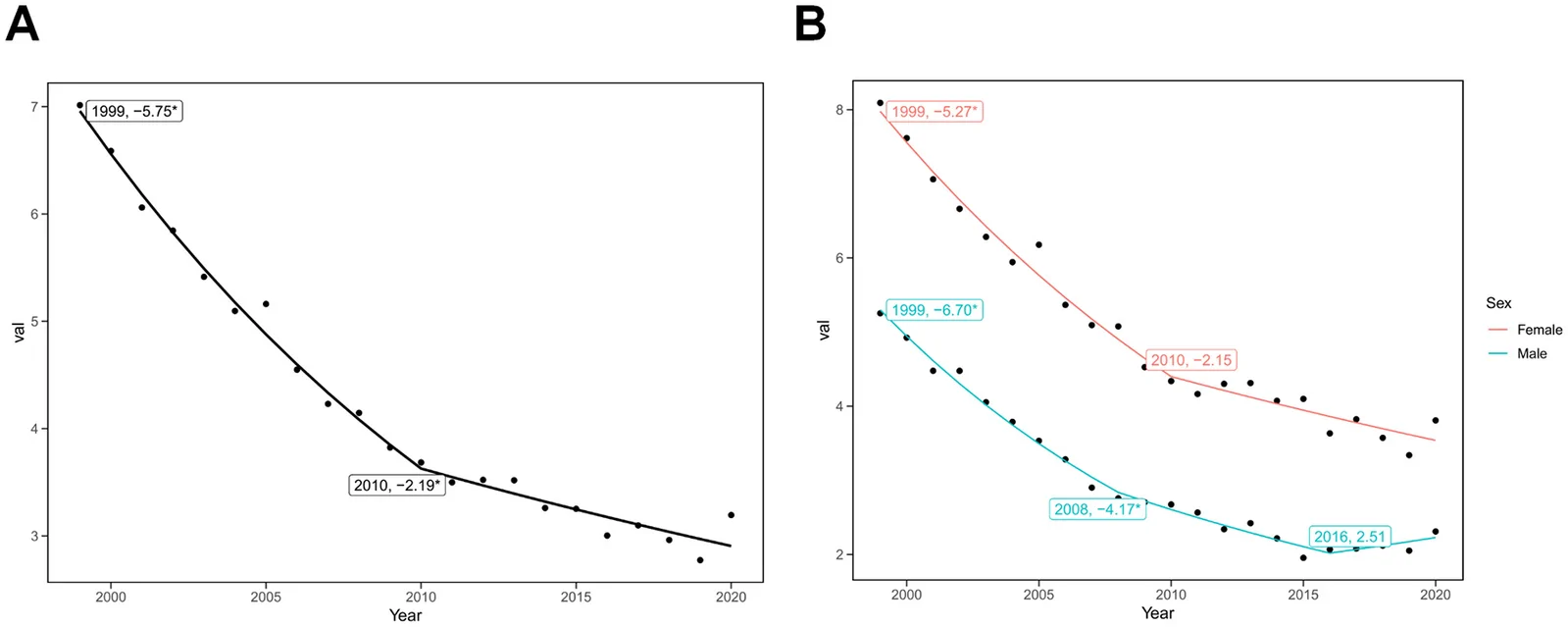
Joinpoint regression analysis of asthma age-adjusted mortality rate trends, 1999–2020. **(A)** Overall. **(B)** Stratified by sex. APC, annual percent change.

Joinpoint regression identified distinct temporal patterns in the overall, female, and male AAMRs. In the overall population, one joinpoint was identified in 2010. The AAMR declined significantly by 5.751% per year during 1999–2010 (95% CI, −6.570% to −5.191%; *P* < 0.001), followed by a slower but still statistically significant decline of 2.194% per year during 2010–2020 (95% CI, −2.944% to −1.029%; *P* = 0.006; Figure 5 and Supplementary Table 4).

Among females, one joinpoint was also identified in 2010. Female AAMR declined significantly during 1999–2010, with an APC of −5.265% (95% CI, −7.192% to −4.500%; *P* < 0.001). During 2010–2020, the estimated decline slowed to −2.153% per year and was no longer statistically significant (95% CI, −3.178% to 0.441%; *P* = 0.067).

In contrast, the male trend contained two joinpoints, in 2008 and 2016. Male AAMR declined rapidly during 1999–2008, with an APC of −6.704% (95% CI, −8.681% to −5.998%; *P* < 0.001). The decline continued during 2008–2016 but slowed to −4.175% per year (95% CI, −5.549% to −2.002%; *P* = 0.004). During 2016–2020, the APC became positive at + 2.513% but did not reach statistical significance (95% CI, −0.057% to 7.902%; *P* = 0.058), suggesting that the previous decline had ceased and that male mortality had entered a plateau or possible upward phase.

Thus, the deceleration observed after 2010 in the overall population should not be interpreted as a uniform sex-specific turning point. The slowdown began earlier among males, in 2008, whereas the corresponding joinpoint among females occurred in 2010.

## ARIMA model selection and diagnostics

Among candidate models evaluated using the BIC, ARIMA (2, 2, 0) was selected as the optimal model for the overall annual AAMR series. The model had AIC = −3.266, AICc = −1.766, and BIC = −0.279. Observed and fitted values were strongly correlated (Pearson's r = 0.991; *P* < 0.0001). The residual Q–Q plot indicated approximate normality, and residual ACF and PACF plots showed no significant remaining autocorrelation. The Ljung–Box test was not statistically significant (Q = 3.392; *P* = 0.335), supporting the conclusion that the residuals were consistent with white noise. Detailed model-selection and diagnostic results are presented in Supplementary Table 6.

## Projections of future asthma mortality trajectories

**Figure 6:**
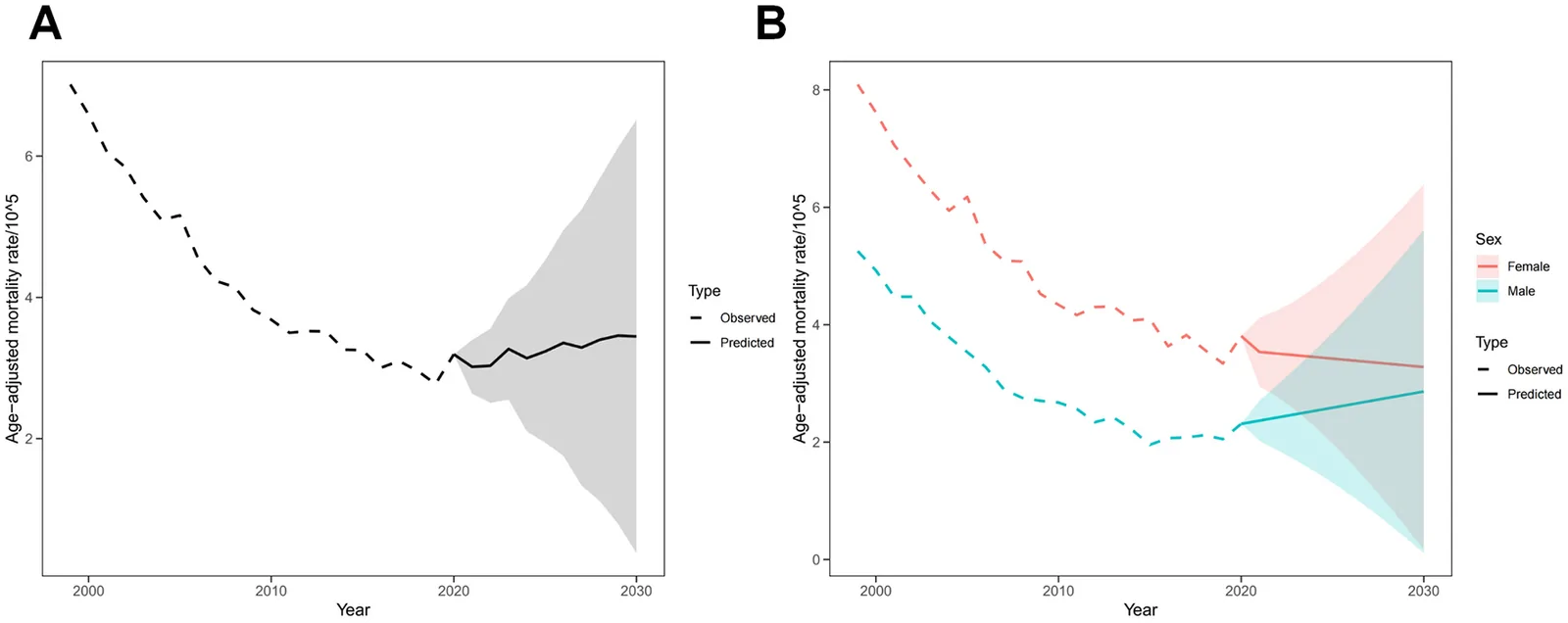
Observed and ARIMA-predicted asthma age-adjusted mortality rates through 2030. **(A)** Overall. **(B)** Stratified by sex. Shaded areas represent 95% prediction intervals.

Under continuation of the historical temporal structure, overall AAMR point estimates were projected to range from 3.02 to 3.46 per 100,000 during 2021–2030 (Figure 6A and Supplementary Table 5). By 2030, the overall point estimate was 3.45 per 100,000, with a 95% prediction interval of 0.38–6.51. Female rates were projected to decline gradually from 3.54 per 100,000 in 2021 to 3.28 in 2030, whereas male rates were projected to rise from 2.37 to 2.86 per 100,000, resulting in progressive narrowing of the sex-specific rates (Figure 6B). These estimates are conditional statistical projections rather than forecasts incorporating future demographic, clinical, environmental, or policy changes.

## Socio-demographic associations and mortality frontiers

**Figure 7:**
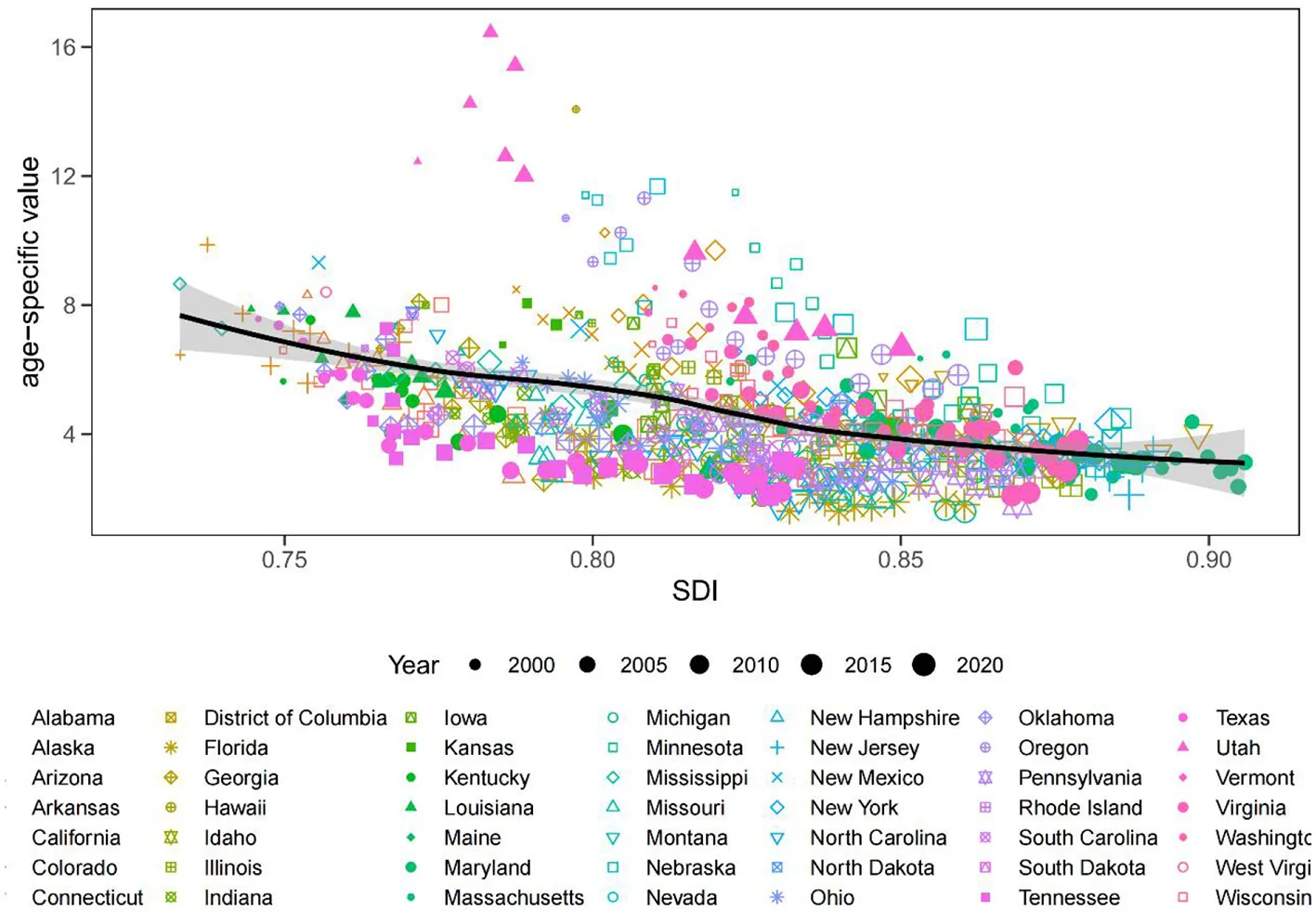
Distribution of disease burden across different SDI levels.

Across the study period, an inverse ecological association was observed between state-level SDI and asthma mortality among older adults. States with higher SDI values generally had lower AAMRs, whereas states with lower SDI values generally had higher AAMRs. The fitted regression line declined as SDI increased from approximately 0.75 to 0.90 (Figure 7). This state-level correlation does not indicate that socioeconomic conditions directly caused differences in mortality and should not be extrapolated to individuals.

**Figure 8:**
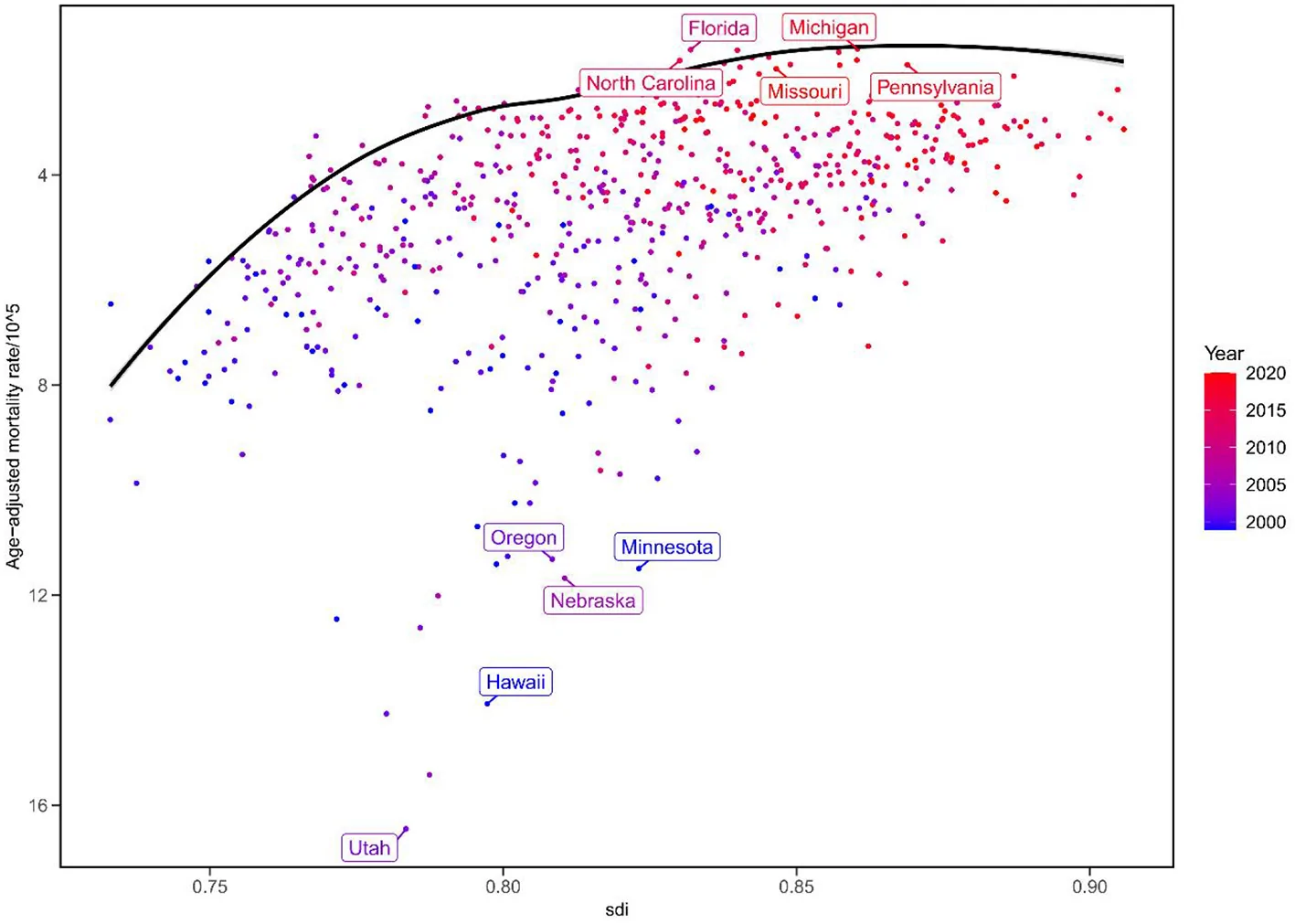
LOESS-based SDI-conditioned frontier of state-level Socio-demographic Index (SDI) and age-adjusted asthma mortality rates (AAMRs) per 100,000, 2000–2020. The frontier represents an empirical population-level mortality benchmark; distance from the frontier is a descriptive gap and not a causal measure of health-system inefficiency or preventable deaths.

The LOESS-based frontier analysis identified substantial variation in mortality relative to the empirical benchmark across the observed SDI range (Figure 8). Florida, North Carolina, Michigan, Missouri, and Pennsylvania were located on or near the estimated frontier, indicating comparatively lower observed mortality than other states with similar SDI values. Hawaii, Minnesota, Oregon, and Nebraska were located farther from the estimated frontier, indicating comparatively larger population-level gaps. Proximity to or distance from the frontier should not be interpreted as direct evidence of healthcare-system efficiency or as a causal estimate of preventable deaths because environmental, demographic, healthcare-access, and measurement-related factors may also contribute.

## Discussion

This study characterized asthma mortality trends among U.S. adults aged ≥65 years from 1999 to 2020 and identified a 54.5% decline in AAMR over the study period. The observed downward trajectory was consistent with previous reports of improving asthma outcomes in the general population and occurred during a period that also included advances in controller therapy, declining smoking prevalence, and expanded public health programs (19–22). However, the ecological nature of the data does not allow us to determine whether these concurrent changes were responsible for the reduction in mortality.

The decline in AAMR slowed after 2010, with the overall APC changing from −5.751% during 1999–2010 to −2.194% during 2010–2020. Women had higher mortality rates than men throughout the study period, although the absolute difference narrowed by 2020. These sex-specific patterns may be consistent with previously reported differences in late-onset asthma, comorbidities, clinical presentation, and healthcare utilization (23–25). The present study did not include individual-level clinical, hormonal, behavioral, or healthcare data and therefore could not determine which factors accounted for the observed differences.

Asthma in later life remains an important and frequently underrecognized clinical challenge. Older adults with asthma may have lower lung function and poorer health-related quality of life than age-matched controls (5), while atypical symptoms, multimorbidity, and overlap with heart failure, COPD, pulmonary fibrosis, gastroesophageal reflux disease, anxiety, and depression can complicate diagnosis (26, 27). Reduced physical activity may mask exertional symptoms (28), and cognitive impairment, impaired dexterity, complex inhaler technique, depression, and medication costs may interfere with adherence to controller and rescue treatment (29). These characteristics provide clinically plausible context for the persistent mortality burden but were not directly measured in the present study.

An important consideration in interpreting these long-term mortality trends is potential diagnostic and coding misclassification between asthma and COPD in older adults. Distinguishing severe or persistent asthma from COPD can be challenging because both conditions may present with chronic respiratory symptoms and persistent airflow limitation, and the two diseases may coexist. Current GINA recommendations emphasize that asthma and COPD are distinct diseases that may coexist, with the term asthma + COPD used to describe patients with features of both conditions rather than a single distinct entity previously conceptualized as asthma-COPD overlap (1). Because CDC WONDER records are based on death-certificate coding with ICD-10 codes J45–J46 and do not contain spirometry, bronchodilator reversibility, inflammatory phenotypes, or other biomarkers, deaths attributed to asthma may have been misclassified or may have occurred in individuals with concomitant COPD (30). Conversely, asthma-related mortality may have been attributed primarily to COPD in some older individuals. Changes in diagnostic concepts and coding practices may therefore have influenced longitudinal comparability. The 2010 joinpoint should be interpreted as a statistical change in mortality trajectory rather than evidence of a specific diagnostic or coding transition.

The deceleration in the overall mortality decline after 2010 should not be interpreted solely as evidence of insufficient public health or clinical intervention. It may also partially reflect an epidemiological floor effect following substantial reductions during the preceding decade. As mortality falls from a relatively high baseline, remaining fatal events may become increasingly concentrated among patients with severe or treatment-refractory asthma, advanced frailty, multimorbidity, and limited cardiopulmonary reserve. In older adults, coexisting cardiovascular disease, chronic heart failure, COPD, metabolic disorders, cognitive impairment, and functional limitations may reduce resilience during acute exacerbations and constrain the extent to which mortality can be further lowered.

Although age adjustment accounts for changes in the overall age distribution, it does not fully capture changes in frailty, disease severity, multimorbidity, or physiological reserve within individual age strata. Consequently, a slower APC at lower mortality levels may represent natural epidemiological stabilization in which each additional reduction becomes progressively more difficult to achieve. At the same time, this interpretation should not obscure potentially modifiable contributors, including underdiagnosis, poor inhaler technique, inadequate adherence to inhaled corticosteroid-containing therapy, delayed recognition of exacerbations, unequal access to specialist and emergency care, and environmental exposures.

Because the present analysis lacked individual-level data on asthma severity, cardiovascular and metabolic comorbidities, frailty, treatment adherence, and end-of-life status, it could not directly quantify the contribution of a floor effect. This explanation should therefore be regarded as a plausible epidemiological interpretation rather than a demonstrated causal mechanism.

Geographic and socioeconomic variation was a prominent feature of asthma mortality in this population. Mortality rates were higher in the West Census Region than in the South and Northeast, and Utah had the highest state-level AAMR. These geographic patterns may be associated with differences in environmental exposure, altitude, population composition, healthcare access, diagnostic practices, death-certificate coding, or other unmeasured state-level characteristics. Although pollen exposure, wildfire smoke, and altitude-related conditions have been discussed as potentially relevant factors in previous studies (31, 32), the present analysis did not directly measure these exposures and cannot attribute regional differences to them.

Similarly, the comparatively lower rates observed in Florida and parts of the Southeast should not be interpreted as evidence that climate conditions, geriatric services, or specific state policies caused lower mortality because these factors were not directly evaluated.

Black and Asian/Pacific Islander older adults had higher AAMRs than White older adults. These disparities were observed alongside documented differences in healthcare access, medication use, environmental exposure, socioeconomic conditions, and structural factors reported in previous research (33, 34). However, the aggregated data cannot determine the relative contribution of these characteristics or establish causal pathways underlying the observed racial and ethnic differences.

Recent surveillance data provide important context for the post-2020 period. Ding et al. (35) reported that asthma was implicated in 3,624 deaths in the United States in 2023, of which 45.9% occurred among adults aged ≥65 years, indicating that older adults continued to account for a substantial proportion of the national asthma mortality burden. These observations reinforce the public health relevance of the plateau projected in our analysis.

However, post-2020 data are distributed through the CDC WONDER single-race database, whereas the primary historical analyses in this study were based on the bridged-race database. The transition changes the treatment of multiple-race decedents, separates Asian and Native Hawaiian or Other Pacific Islander populations, and introduces a different race-specific denominator framework. Direct concatenation of these race-specific series could therefore compromise longitudinal comparability.

An additional clinical dimension not captured in the present analysis is place of death. National studies have evaluated asthma deaths among older adults across hospitals, private residences, nursing homes, hospice facilities, and other settings (35). Complementary U.S. death-certificate data from 2000 to 2019 showed that, although the overall asthma mortality rate declined by approximately 32%, the at-home mortality rate remained essentially unchanged, from 0.32 to 0.34 per 100,000; consequently, the proportion of asthma deaths occurring at home increased from approximately 23% in 2000–2001 to 36% in 2018–2019 (36). These observations indicate that reductions in hospital-based mortality may coexist with persistent fatal events outside medical facilities.

This pattern may be particularly relevant to older adults, in whom cognitive impairment, reduced manual dexterity, poor hand–lung coordination, multimorbidity, polypharmacy, medication costs, social isolation, and dependence on caregivers can interfere with controller adherence and correct inhaler use. Delayed recognition of worsening symptoms and delayed access to emergency services may allow a severe exacerbation to progress before hospital treatment is initiated. Current asthma-management strategies emphasize inhaled corticosteroid-containing treatment, regular assessment of adherence and inhaler technique, prompt treatment escalation, patient and caregiver education, and individualized written action plans (1).

Nevertheless, death at home should not automatically be classified as preventable or as evidence of inadequate healthcare. Mortality records cannot reliably distinguish sudden unexpected exacerbations from planned home or hospice deaths and do not provide information on prescribing, adherence, inhaler technique, caregiver involvement, or patient preferences. Future studies linking mortality records with healthcare-utilization and prescription data may help determine whether persistent out-of-hospital mortality reflects gaps in asthma control, barriers to emergency access, limitations in transitional care, or end-of-life preferences.

Our ARIMA projections should be interpreted in the context of broader demographic forecasting frameworks. GBD-based analyses commonly use age-period-cohort or Bayesian age-period-cohort models together with age-specific disease estimates and projected population structures (37). These approaches can distinguish changes in epidemiological rates from increases in absolute burden associated with population growth and aging. Among adults aged ≥70 years, absolute COPD deaths and disability-adjusted life-years increased despite declining corresponding rates (38). A recent analysis of major chronic respiratory diseases among adults aged ≥55 years used Bayesian age-period-cohort and Nordpred models to incorporate age, period, cohort, and demographic information (39).

In contrast, the present ARIMA model was designed for conditional extrapolation of a single national annual AAMR series over a ten-year horizon. Its parsimonious structure is appropriate for characterizing temporal dependence in the 22 annual observations but does not explicitly represent population aging, birth-cohort effects, competing causes of death, environmental exposures, treatment uptake, healthcare access, policy interventions, or unexpected shocks. Therefore, projected stabilization of AAMR at approximately 3.02–3.46 per 100,000 should not be interpreted as stabilization of the absolute mortality burden. The number of deaths may still increase as the population aged ≥65 years grows. These projections should be viewed as short- to medium-term trend-continuation scenarios rather than structural forecasts of future disease burden.

The SDI-conditioned LOESS frontier identified substantial state-level variation relative to an empirical mortality benchmark. Some states had lower observed mortality than other states with comparable SDI values, whereas others had larger frontier gaps. These findings may help identify settings for further investigation but do not demonstrate that particular resource-allocation strategies, healthcare programs, or policies caused lower mortality. The frontier gap may reflect air pollution, pollen exposure, wildfire smoke, climate and altitude, urbanization, insurance coverage, specialist access, healthcare infrastructure, treatment adherence, diagnostic practices, death-certificate coding, or other unmeasured factors.

Accordingly, proximity to the frontier should not be interpreted as superior healthcare-system efficiency, and distance from the frontier should not be interpreted as a direct estimate of preventable deaths. Individual-level studies and appropriately designed evaluations of healthcare and public health interventions would be required to test these hypotheses.

These findings extend earlier CDC WONDER-based studies by combining Joinpoint trend characterization, transparent ARIMA diagnostics, and SDI-conditioned population-level benchmarking. The integrated approach provides complementary descriptive perspectives, while each method remains subject to the assumptions and limitations described above.

Despite these strengths, several limitations warrant consideration. First, the analysis relied on death-certificate-based electronic mortality records. As highlighted in epidemiological studies using routinely collected administrative health data, such records may contain incomplete or inaccurate information and may lead to underascertainment or misclassification (10). This concern is particularly relevant in older adults, among whom asthma may be underdiagnosed or attributed to COPD (30).

Second, all analyses were based on aggregated population-level data. Associations observed across states, demographic groups, or time periods may not reflect individual-level associations, and interpreting them as such would risk ecological fallacy. Potentially relevant variables, including disease severity, smoking history, medication adherence, comorbidities, insurance status, environmental exposure, healthcare access, and specialist availability, were unavailable; residual and unmeasured confounding may therefore account for some observed associations.

Finally, the LOESS frontier is a relative statistical benchmark rather than a causal estimate of healthcare performance or preventable mortality. ARIMA projections assume continuation of historical patterns and cannot account for major unforeseen events, changes in clinical practice, environmental shocks, or policy interventions. Future studies incorporating post-pandemic data and individual-level cohorts, such as the National Health and Nutrition Examination Survey, are needed to validate these population-level trends and improve projection models.

## Conclusion

From 1999 to 2020, the AAMR of asthma among U.S. adults aged ≥65 years declined by 54.5%, although the rate of decline slowed substantially after 2010 and appeared to plateau among men after 2016. Marked differences persisted by sex, race/ethnicity, geographic region, and state-level socioeconomic conditions. ARIMA projections suggest that AAMR may remain relatively stable through 2030. If these rates coincide with continued population aging, the absolute number of asthma-related deaths may remain substantial. These findings identify population-level patterns that warrant continued surveillance and further investigation. Because the study was based on aggregated observational data, the associations should not be interpreted as evidence that demographic, socioeconomic, environmental, healthcare, or policy factors caused the observed mortality patterns.

## Data Availability

The original contributions presented in the study are included in the article/Supplementary material, further inquiries can be directed to the corresponding author.
