# Supplementary material for: Declining yet persistent asthma mortality among U.S. adults aged 65 years and older (1999–2020)
Source: Front Med (Lausanne). 2026 Aug 21;13:1832774. doi: 10.3389/fmed.2026.1832774 (PMC13542643; doi:10.3389/fmed.2026.1832774)
Supplement: Supplementary file 1 [file Data_Sheet_1.pdf]

Supplementary Material

1 Supplementary Methods

This appendix aims to provide a detailed account of how each data source used in the analysis was identified, retrieved and handled. Its purpose is twofold. First, to allow readers to assess the evidence on which I base the analysis and argument; second, so that the conditions of access to the research participants are themselves transparent and significant, as discussed in the Metadata section of the article. The sources are henceforth listed and organized by type, archival documents, interviews and supplementary fieldwork data, and presented in the order in which they are discussed in the article itself. Thereafter, a discussion of how the word-frequency analysis (see Table 1 and Figure 1) is provided, before closing with a discussion of the ethical considerations that informed the research before, during and after fieldwork.

2. Archival Documents

2.1. List of Sources

- Omerdić, M. (1992) 'Fatwa on Raped Women', *Glasnik Rijaseta Islamske zajednice*, 3-4, pp. 168-169. [Archival Source 1]

- Omerdić, M. (1992) 'On Chetniks' Crimes Committed over Muslim Girls and Women', *Oslobođenje*. [Archival Source 2].

- Omerdić, M. (1993) 'Chetnik Crimes Against Muslim Women: It's not the children's fault!', *Oslobođenje*, 18 January. [Archival Source 3]

- Nurudin, D.A. (1993) 'A Message to Raped Women' [Fatwa]. *Islamic Thought*. [Archival Document 4]

- Islamic Community of BiH (2023) 'Sermon on Sexual Violence in War' [Sermon delivered on the occasion of the International Day of Combating Sexual Violence in War]. [Archival Document 5].

- Interreligious Council of Bosnia and Herzegovina (2019) *Handbook for Religious Professionals for Work with Victims of Rape in War*. [Archival Document 6].

2.2. Retrieval

The archival corpus includes six documents produced by, or in collaboration with, the Islamic Community of Bosnia and Herzegovina (IC) between 1992 and 2023. These sources were not retrieved in advance of fieldwork but obtained through interview-driven elicitation, as all of them were identified and retrieved thanks to the interviewees themselves.

The four wartime fatwas (Archival Document 1-4) were issued by elite representatives of the IC between 1992 and 1993 in direct response to the sexual violence being perpetrated against the Bosniak Muslim population. After their publication, they gained considerable national and international traction and some of them, like Archival Source 1, were cited in scholarship on religion and transitional justice (e.g. Spahic-Siljak and Anic, 2022). Despite this, they were still not the most straightforward to locate.

Digitalised copies that had the entire transcript were held at the Gazi Husrev-beg Library in Sarajevo and were ultimately provided by the elite respondent from the Riyasat, as the topic of fatwas did come up during out interview. All documents were in Bosnian, with the exception of Archival Document 3. For the purpose of the discourse analysis, the former were first translated using DeepL translator, the most accurate translator to date, and then cross-referenced with a research assistant, a native Bosnian speaker.

Archival 5, the khutbah "Sexual Violence in War" delivered during Friday prayer in June 2023, was not publicly available online despite the IC maintaining a digital archive of sermons. Its existence came to my knowledge during conversations with the same elite respondent, who provided a digitalised version of it.

Archival 6, the *Handbook for Religious Professionals for Work with Victims of Rape in War* (2019), was produced by the Interreligious Council of BiH in collaboration with the IC, as well as the other three religious denominations in the country – Orthodox Church, Catholic Church and the Jewish community - UNFPA, and the British Embassy. A physical copy was first mentioned by one of the imams interviewed and obtained subsequently from a representative of the Interreligious Council in Sarajevo after his recommendation. At the time of writing, the Handbook is not available through any publicly accessible institutional website. It is, to the best of my knowledge, the only guidance document the IC has produced for its leaders on how to engage pastorally with CRSV and those it has affected. That both the khutbah and the Handbook emerged organically from the interviews rather than from prior desk research.

2.3. Word Frequency Analysis

The word frequency analysis shown in Table 3 was conducted in three steps. First, using the software NVivo, the search identified the gender-specific language across the corpus. Thereafter, since no targeted filter could be applied directly, the results were reviewed manually by me to ensure that the terms were counted only when referring to those affected by conflict-related sexual violence, thus excluding generic references to individuals not directly implicated as victims-/survivors. Finally, frequency counts were then normalized to allow comparison across sources of different lengths, expressed as occurrences per 1,000 words: (raw count / total word count) x 1000. The terms examined were: *woman/women; girl(s), mother(s), female(s), daughter(s), sister(s), wife/wives, she/her*, for female-specific language; and *man/men, boy(s), father(s), male(s), son(s), brother(s), husband(s), he/his* for male-specific language.

The word cloud presented in Figure 1 was produced through a similar two-step process. The corpus was first filtered manually to retain only phrases that referred to victims-/survivors and contained descriptive adjectives. These filtered excerpts were then processed in NVivo through a word frequency analysis, which could then generate the word cloud presented in this article. This approach was necessary to prevent adjectives from being conflated with verb forms (e.g. rape) or with descriptive language referring to contexts or actors that were not the victims-/survivors themselves.

Because grammatical structures make it possible to discern the function of each word and the subject of each sentence, ambiguous cases were rare and mostly involved words that could function as both an adjective and a verb (e.g. raped; “the raped woman” vs “she was raped”).

3. Interviews

3.1. List of Respondents

Elite and Local Religious Leaders of the Islamic Community

- Elite Religious Leader, Sarajevo, March 2025.
- Imam, Sarajevo Canton, December 2025.
- Imam, Brčko District, December 2025.
- Imam, Central Bosnia Canton, January 2026.

Civil Society Organizations

- Representative, Association A, Sarajevo, April 2025
- Representative, Association B, Sarajevo, March 2025.

3.2. Sampling

Participants within the Islamic Community were identified through purposive sampling. Initial contact was made with two representatives within the Riyasat, the executive body of the Islamic Community: a man and a woman, both of whom held administrative roles relevant to the research topic. The female representative agreed to participate and, following the interview, facilitated introductions to religious leaders at lower institutional tiers. This snowballing approach, while standard practice in research involving sensitive topics and closed institutional settings, means that the sample is not independent of elite mediation. The three imams who participated were introduced either directly by the elite representative, none was approached without prior knowledge of the research having been communicated through this channel. Hence, access was dependent on institutional gatekeeping, and it is likely that participants whose views diverge most sharply from the official line of the IC were among those least reachable through a recruitment path originating from within the Riyasat. The analysis is alert to this constraint and does not claim to represent the full range of positions within the institution.

Participants that represented civil society organisations were also identified through purposive sampling, through the criterion of being non-governmental organisations that worked in Bosnia and Herzegovina on the topic of wartime sexual violence. Their contacts were found throught their respective website.

3.3. Interview Guide

The interview guides were developed prior to the start of fieldwork, allowing time for consideration of what was being asked, as well as how the questions related to both existing research and findings within the field of wartime sexual violence and gender-based violence more generally. Because this research article is part of a larger project on how communities respond to the phenomenon of conflict-related sexual violence, four different interview guides were drafted, asking very similar questions but calibrated towards different respondents, being religious leaders, civil society organisations, community members and victims-/survivors of CRSV.

To ensure linguistic and conceptual accuracy, the guide went through back-translation, from English to Bosnian to English. This process was possible thanks to a team of two research assistants. The script was then discussed with them in two separate occasions before a final version was agreed upon. The script was also rehearsed before fieldwork with my interpreter, who was one of the research assistants involved in the back-translation process.

The research assistants were native Bosnian speakers and professionals within the field of peace and conflict studies and holocaust and genocide studies respectively. This allowed the questions to be reflected upon while being contextualized socially, politically and culturally. Hence, the research assistants' background was key in this process and created space for us to discuss cultural sensitivity and power dynamics, all of which strengthened the interview guide. In doing so, a balance was reached between technical equivalence – literal translation – and conceptual equivalence – translation that is grounded in context and meaning.

Interview questions were organised around two thematic clusters: participants' understanding of conflict-related sexual violence as a phenomenon, and their personal and institutional engagement with victims and survivors. Questions were deliberately framed in gender-neutral language throughout, a choice that required particular care in Bosnian, given the grammatical gender of key terms. Neither the word for "victim" (*žrtva*, feminine) nor the word for "survivor" (*preživjeli*, masculine) is gender-neutral in Bosnian; participants were instead referred to as "persons who were affected by this violence" (*osobe koje su bile pogodjene ovim nasiljem*) until the point at which they were directly asked whom they had in mind when using these terms. Equally, given the research question at the core of this article, questions were careful never to specify or default to a particular form of harm, such as rape, instead speaking of wartime sexual violence in general terms. This allowed respondents to define the phenomenon on their own terms, and permitted their implicit assumptions about both the nature of the violence and the identity of those it affected to surface without being prompted.

Moreover, questions were never asked in relation to one's victimisation or experience of sexual violence specifically during to war, an aspect of the interviews which I discuss in Section 4.1. of this appendix.

3.4. Interview Procedure

Six total semi-structured interviews were conducted with representatives of the Islamic Community of Bosnia and Herzegovina and civil society organizations with headquarters in Sarajevo between March 2025 and January 2026. All interviews were conducted in person except for one, that with the imam of Central Bosnian Canton, for which questions were sent and answered digitally using an end-to-end encrypted messaging app.

Most interviews were conducted in Bosnian with the assistance of an interpreter who provided consecutive interpretation[1] during the in-person interviews and translated the written interview. The in-person interviews were recorded with the consent of the interviewees and transcribed by me using MacWhisper. This software automatically translated the parts of the interviews conducted in the local

[1] This differs from simultaneous interpretation in that consecutive interpretation consists of the translations of segments of a conversation during which the speaker pauses periodically to allow the interpreter to render the segment after each pause; whereas simultaneous interpretation happens concurrently as the respondent speaks.

language, allowing the interpreter's translation to be reviewed for accuracy against the Bosnian transcript. This is a key step given the methodology chosen to answer the research question, as FCDA centers language and terminology at the core of the inquiry. All interviews were then reviewed to omit identifying information, before both the recording and the unclassified transcript were deleted. All data is stored in a double-password protected device to which only I have access.

3.5. Coding of Interviews

The interviews were coded thematically on Nvivo to identify the four main themes on which the analysis relies. These were then analysed manually by me, as critical discourse analysis as a technique requires more granular attention to the language used and the ways it is used.

4. Ethical Considerations

4.1. Ethical aspects of the interview guide

The interview guide was drafted similarly but questions were adjusted depending on the target respondents (religious leaders, civil society organisations, community members and victims-/survivors). Because of the extent to which people were victimised during the war in Bosnia and Herzegovina in the 1990s, and because the research objective of the project to which this research belongs does not investigate the causes, dynamics or experiences of this violence, no question was asked in relation to one's wartime experiences. The interview guide was deliberately drafted to focus on post-war community dynamics, with particular attention to how one would understand societal responses to the phenomenon of wartime sexual violence and the role that religious leaders played and play in supporting those affected.

Although this research took a community-centred approach rather than a survivor-centred approach, to avoid research fatigue for victims-/survivors of wartime sexual violence that had been consistently voiced during my first fieldwork trip to Bosnia and Herzegovina, the drafting of the interview guide for the victims-/survivors was a key exercise for those interviews with NGO representatives that had experienced this violence themselves. This meant that, upon acknowledgment of their experiences, questions that for instance, were meant to get insights on how one understands the phenomenon of wartime sexual violence (e.g. if as only rape or encompassing different forms) or of how one thinks of victims-/survivors (e.g. if only female or male) were not asked. Instead, the conversations pivoted solely around the work of the association and how they see the role of religious leaders in helping them with their endeavours and in helping the wider community in navigating their lives after war.

This does not mean that the topics of discussion were not sensitive and potentially re-traumatising for some. Because of this, I strove to keep the conversation focused on community responses and support measures where I could, while also making space for respondents to share their own experiences of victimisation if and when they chose to. Having a semi-structured interview guide was key in this regard, as it allowed flexibility during our conversations, avoiding these to ever feel mechanic. I thus never interrupted the respondents when they wanted to share experiences of violence where they felt these were important parts of their answers, respecting their agency in choosing whether and to what extent to share. Once harder stories of victimisation were shared, I acknowledged them and returned slowly to the topic at focus, never in an abrupt manner.

4.2. Ethical aspects of involving human participants

This research was approved by the Swedish Ethical Review Authority, with Application Number 2024-02253-01.

Participants were always approached with open and transparent communication about what the interviews were going to focus on. The participants were asked to choose the time and place of the interview so that this would feel most comfortable for them. Upon arrival, both my interpreter and I re-introduced ourselves and went through the project and the interview procedure. Participants were given a consent form and were asked to give verbal consent to ensure their anonymity. They were then welcomed to keep the consent form, which included information about data protection as well as contacts of my supervisor back at Uppsala University, my interpreter and me.

Prior to the start of the recording, participants were informed of their right to withdraw at any point and to decline to answer any question; no participant exercised either right in practice. Participants were also told during the consent process that quotations might be used and were allowed to request that specific statements not be quoted directly. Some respondents did, for some parts of their interviews, exercise this right. Upon them signalling such desire, the recording was stopped altogether, or the said part was redacted from the final transcript. All participants are referred to by institutional designation throughout the text; no identifying information is included in the analysis, the footnotes, or this appendix. The archival documents are cited in full in the reference list and are treated as institutional rather than personal sources.

Trust was a red thread that ran throughout the interviews and beyond. While respondents like the elite religious leaders were only interviewed once, my interpreter and I met with her on two other separate occasions during both my first trip to Bosnia and Herzegovina between March-April 2025 and between November-December 2025. This allowed the snowballing strategy to reach local religious leaders to unfold.

Given the small sample involved in this study, particularly when it comes to religious leaders holding specific institutional positions in a relatively small country like BiH, confidentiality did not rely on pseudonymisation alone, as institutional designation, gender and geographic post could still make individuals identifiable to a knowledgeable reader. To manage this, I aggregated geographic references where location's precision was not analytically necessary (e.g. Central Bosnia Canton; Brčko District, Sarajevo Canton) and altered secondary identifying information such as exact interview dates, both in the transcripts and in my notes. While this proved relatively straightforward for grassroots religious leaders such as imams, elite religious leaders at the level of the Riyasat could be more easily identified, also given the fewer positions available to women. Hence, while the same precautions were adopted, the level of institutional specificity that could be shared was agreed with participants in advance. In this specific case, the respondent not only stated to be comfortable with this and her answers – whose transcript was also provided to her before the data was processed - but was also inclined to have her full name shared. While full disclosure was not considered appropriate, the details that appear in the text have been directly agreed upon by the respondent herself.

4.3. Ethical aspects of researching CRSV for both the author and the interpreter

Even though interviews dealt with post-war social dynamics rather than experiences of wartime sexual violence, the research itself broadly touched on a very sensitive subject. This meant that care had to be

exercised not only toward respondents, but toward ourselves – my interpreter and I – throughout the interview process and beyond.

First, we made a habit of following each interview with a debrief, where we discussed our impressions and reflections, and made space to give each other feedback. These reflections became so integral in our routine that we are now reworking them into a methodological piece on qualitative interviews. Second, although interviews were spread out across days and weeks, we still needed time to decompress, both from the immediate effort of sustaining and translating a conversation, and from the mental toll that speaking about sensitive topics such as CRSV entails. This held true not only when participants shared direct or indirect experience of victimisation, but also because the sensitivity of the questions required us to constantly calibrate our tone, language and body language throughout the interviews. The ways in which my interpreter and I approached the topic, and our physical demeanour while doing so, did in fact not go unnoticed, with several respondents commenting positively on it. This was hardly incidental, but a result of the time we both deliberately set aside to decompress, whether through movement, friends or other forms of care.

4.4. A note on positionality

A note on positionality is warranted here, and it bears directly on the data that was gathered. I conducted fieldwork as a young non-Muslim woman from outside Bosnia and Herzegovina, without prior professional or personal connections to the institution or the communities affected by the conflict. My research assistant, who supported me with interpretation and logistics, was also a non-Muslim young woman.

This positioning, women asking men about sexual violence, in a context where the subject remains socially and institutionally sensitive, may have shaped the research in ways that are important to acknowledge. For instance, it is possible that if, on the one hand, it facilitated a degree of openness from the female elite religious leader, on the other it might have contributed to a specific framing of the subject from the male religious leaders. In other words, speaking to two young women about sexual violence might have led to particular attention on the part of the respondent to orient the conversation towards women as the primary victims of this violence. Whether the relative absence of discussion of male survivors in the interview data reflects the respondents' own assumptions, the implicit signals carried by the researchers, or both, cannot be determined with certainty; but the question is worth holding.
